# The Impact of Mobile Health Use on the Self-care of Patients With Type 2 Diabetes: Protocol for a Randomized Controlled Trial

**DOI:** 10.2196/31652

**Published:** 2022-06-17

**Authors:** Lucija Gosak, Majda Pajnkihar, Gregor Stiglic

**Affiliations:** 1 Faculty of Health Sciences University of Maribor Maribor Slovenia

**Keywords:** diabetes, mobile application, disease management, self-care

## Abstract

**Background:**

Chronic diseases are a substantial public health issue worldwide and affect an individual’s quality of life. Due to the alarming rise in type 2 diabetes, health care that was primarily focused on diagnosis and treatment of the disease is increasingly focused on prevention and self-care. Patients who adhere to a constant and strict treatment regimen (physical activity, diet, medication) and regularly monitor their health are more likely to maintain self-care and health, prevent the exacerbation of the disease, and avoid the complications of diabetes (retinopathy, diabetic feet, etc). In recent years, many innovative devices that have become increasingly present in inpatient health care, such as mobile apps, are available to help patients maintain consistency in monitoring their health status. Mobile apps make it easier for individuals to monitor their self-care and illness and follow instructions regarding disease control.

**Objective:**

This study aims to determine the impact of mobile app use on self-care in patients with type 2 diabetes. We will evaluate and test the usefulness of the *forDiabetes* app as a tool to improve the self-care of individuals with type 2 diabetes.

**Methods:**

We will perform a double-blind randomized controlled trial. The study will include individuals aged over 18 years diagnosed with or have regulated type 2 diabetes who are treated in family medicine practices. Additionally, the individuals included in the study should not have any acute complications due to the consequences of type 2 diabetes. They will use an Android or iOS mobile phone and a blood glucose meter during the investigation. With the help of simple randomization, individuals will be divided into the intervention and control groups. Individuals in the intervention group will use the *forDiabetes* mobile app to monitor their self-care for type 2 diabetes. Individuals in the control group will not receive a particular intervention. Data will be collected using the *Self-care of Diabetes Inventory* questionnaire and *Brief Illness Perception Questionnaire.* Blood sugar, blood pressure, glycated hemoglobin (HbA_1c_), and weight measurements will be monitored using calibrated instruments during the study by the nurses employed at the family medicine practice. Data will be collected at the beginning of the study and after a patient visits the family medicine practice.

**Results:**

In the first half of 2020, we have prepared a translation of the mobile app that will be used by the participants of the intervention group, as well as more detailed instructions for using the app. We have also prepared a translation of the questionnaires in Slovene. The research results will be published in 2023.

**Conclusions:**

This research contributes to greater visibility and usability of mobile apps for the self-care of patients with type 2 diabetes and raises awareness of the possible use of innovative methods.

**Trial Registration:**

Clinicaltrials.gov NCT04999189; https://clinicaltrials.gov/ct2/show/NCT04999189

**International Registered Report Identifier (IRRID):**

PRR1-10.2196/31652

## Introduction

### Background

Type 2 diabetes is a complex chronic disease [1,2] with an enormous global burden that has substantial implications for human health [3]. The consequences of the disease cause substantial economic and social responsibilities [4] and pose a growing public health problem [5,6]. Preventing and limiting the progression of the disease is an urgent task worldwide [7] and remains an important challenge for individuals and society [8]. The self-care of type 2 diabetes is key to containing the disease [9,10], reducing disease complications, and improving quality of life [11]. By supporting patients with type 2 diabetes in healthier self-care behaviors, we can influence the outcome of the disease [5]. The constant monitoring of diabetes and lifestyle factors can contribute to better health [12]. Many complications are the result of uncontrolled disease [13].

The current COVID-19 pandemic affects the direction of and interrupts health care, disrupting long-term conditions including type 2 diabetes [14,15]. The need of self-care for patients with type 2 diabetes is increasingly present [16]; therefore, mobile technology has become an essential tool for promoting self-care [17]. The optimal use of mobile technologies is necessary to achieve benefits and optimal cost efficiency [18]. A mobile app strengthens self-care and contributes to better information, health education, and self-confidence in dealing with the disease [19]. Knowledge of mobile apps has been identified as an essential factor enabling independent disease management and self-care [20]. The use of new technology is one way to bridge the gap between patient needs and providing health care [21]. A mobile health app for patients with type 2 diabetes affects their knowledge of the disease, improves blood glucose control [22], and positively affects self-care [23]. In the future, mobile apps will represent an essential part of the health care system, so research in this area is needed [24]. Mobile apps show potential for disseminating self-care education [4]; improving motivation [25], disease symptom management, and patient experience [26-28]; helping patients with strict treatment requirements [29] and the adoption of a healthy lifestyle [30]; and reducing care costs [31-33].

As a guide to the self-care of individuals with type 2 diabetes using the mobile app, we will use the Middle-Range Theory of Self-Care of Chronic Illness. Self-care is a general concept built from 3 key ideas: self-care maintenance, monitoring, and management. Maintaining self-sufficiency (or self-care maintenance) refers to an individual’s behaviors to improve well-being and maintain health and stability. Self-care monitoring refers to the process of observing oneself due to changes in signs and symptoms. Self-care management is defined as responding to signs and symptoms when they occur [34,35].

### Objectives and Hypotheses

Objective 1 is to assess the impact of using the mobile app on the assessment of patient self-care. Hypothesis 1 states that in the intervention group of patients with type 2 diabetes, the self-sufficiency assessment at the first control visit will increase compared to the control group that did not use the mobile app.

Objective 2 is to assess the impact of mobile app use on disease perception. Hypothesis 2 states that in the intervention group of patients with type 2 diabetes, perception at the first control visit will improve compared to the control group that did not use the mobile app.

Objective 3 is to assess the impact of mobile app use on blood sugar and HbA_1c_ levels. Hypothesis 3 states that in the intervention group of patients with type 2 diabetes, blood sugar and HbA_1c_ levels at the first control visit will decrease compared to the control group that did not use the mobile app.

Objective 4 is to assess the impact of using the mobile app on the patient’s body weight. Hypothesis 4 states that in the intervention group of patients with type 2 diabetes, the body weight at the first control visit will decrease compared to the control group that did not use the mobile app.

Objective 5 is to assess the impact of mobile app use on the patient’s blood pressure values. Hypothesis 5 states that in the intervention group of patients with type 2 diabetes, the blood pressure value at the first control visit will decrease compared to the control group that did not use the mobile app.

## Methods

### Randomized Controlled Trial Design

To determine the effectiveness of using the mobile app on the self-care of patients with diabetes, we will conduct a double-blind randomized controlled trial. The study will include people aged over 18 years diagnosed with type 2 diabetes who are treated in family medicine practices in Slovenia. It will also include patients who have already been given some form of treatment to manage the disease and do not have chronic complications due to the disease (retinopathy, diabetic foot, nephropathy, neuropathy, etc). We will ensure this by receiving information on the patient’s health status from the medical staff. The patients will use a mobile phone and a blood glucose meter during the research period. Individuals will use the *forDiabetes* [36] mobile app in the intervention group. *forDiabetes* [36] is an existing app designed for diabetes self-care and allows the tracking and monitoring of crucial diabetes data (physical activity; high-carbohydrate foods consumed; blood glucose, blood pressure, and HbA_1c_ levels; weight; medications; etc). Before selecting the mobile app, its quality was assessed by 2 nurses using the Mobile Application Rating Scale: user version [37]. The mobile app was rated as high quality and suitable for patients (mean 4.54, SD 0.33; on a scale out of 5) by the nurses. In accordance with the American Association of Diabetes Educators, we also assessed whether the mobile app includes self-care behaviors [38]. The mobile app contains behaviors from the awareness of the social determinants of health, integration of technology into self-care, and role of the diabetes care and education specialist.

In the control group, individuals will not use any diabetes technologies to guide the self-care of their disease, but they will be using a blood glucose meter. The control group patients will manage their disease as before and attend scheduled checkups.

Before the start of the study and at the follow-up visit to the family medicine practice after using the mobile app, we will perform assessments of self-care and disease perception using the *Self-care of Diabetes Inventory* (SCODI) [39] and *Brief Illness Perception Questionnaire* (Brief IPQ) [40] instruments, respectively. A nurse employed at the family medicine practice will also monitor blood sugar, blood pressure, and glycated hemoglobin (HbA_1c_) levels and body weight in the intervention and control groups with standardized, validated instruments. The nurse will use the standardized calibration devices according to the guidelines for each measurement. Data will be recorded at each inspection. We will also collect the data that the patients will enter daily into the mobile app (physical activity; high-carbohydrate foods consumed; blood glucose, blood pressure, and HbA_1c_ levels; weight; medication; etc) in real time.

**Figure 1:**
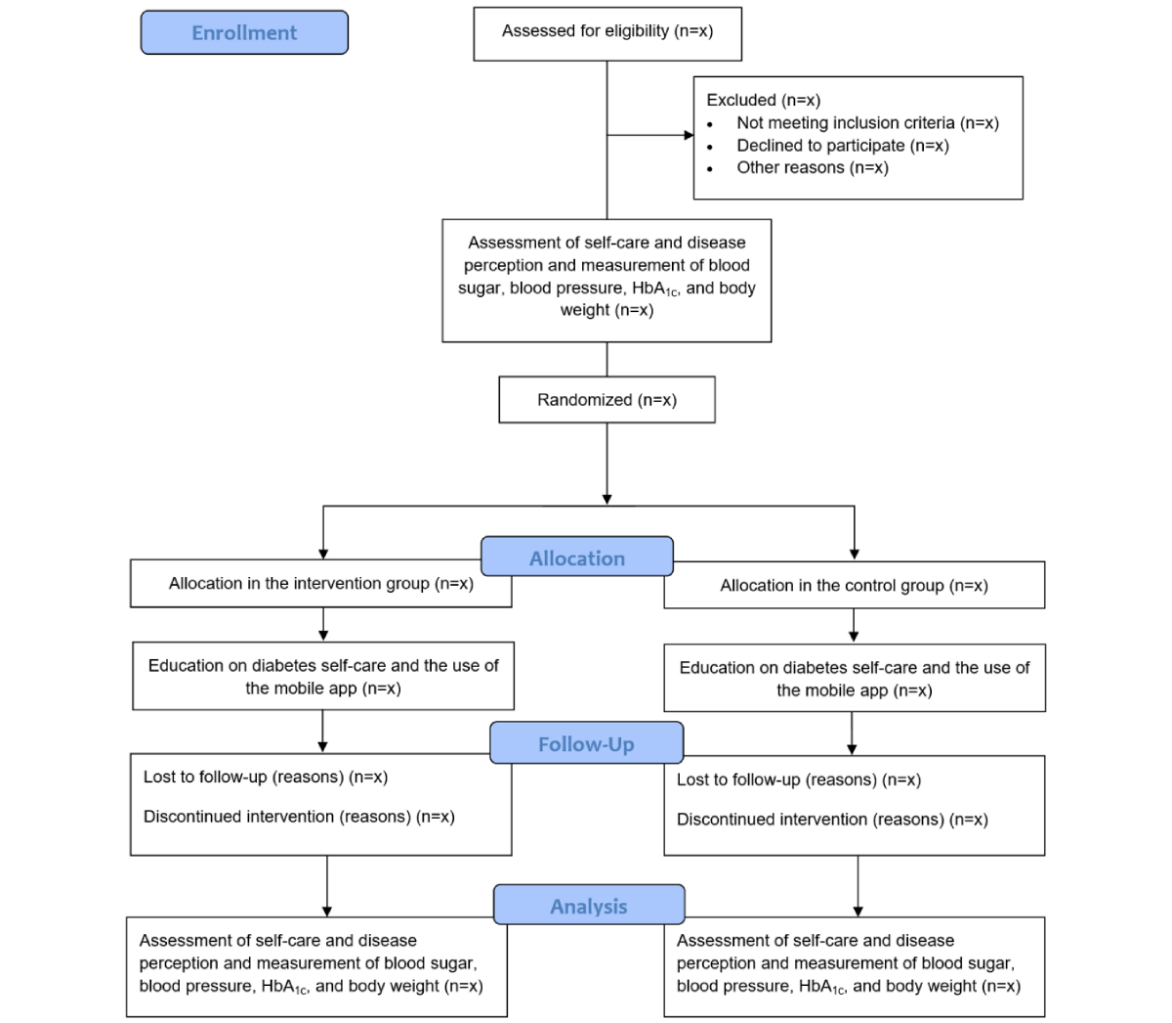
Protocol for conducting a randomized controlled trail. HbA_1c_: glycated hemoglobin.

The research will be carried out in family medicine practices where individuals with type 2 diabetes are treated throughout Slovenia. The study will cover at least one private and one public family medicine practice from each regional unit in Slovenia. We will use a simple randomized approach to select the family medicine practices for cooperation from a preprepared list of family medicine practices. Before conducting the research, we will obtain the necessary consent from all institutions where the study will take place to enter the family medicine practices. We will also get the required permits from the individuals involved. At the beginning of the randomized controlled trial, we will obtain the written or oral consent of the institutions to participate in the research and enter their environment. The institutions will be provided with all necessary information and the research purpose. In the introductory part of the research, the participants in the study will be informed about the purpose and goal of the study, ensuring the anonymity of the participants, the voluntary nature of participation, and the possibility of withdrawing from participation. The survey will run for 1 year. Figure 1 presents a graphical illustration of the research process.

### Mobile App Translation

The mobile app *forDiabetes* [36] includes the required self-care behaviors according to the American Association of Diabetes Educators [38] and is suitable for maintaining self-care in individuals with type 2 diabetes. Before starting the research, we have obtained written consent to use the mobile app. *forDiabetes* [36] is an existing app translated from English into Slovene before the research was conducted. The text was translated by 2 researchers who have the necessary knowledge in self-care and English. In the case of wording deviations, there was a discussion between the researchers to find a Slovene word as close as possible to the meaning of the English word and used as professional terminology in the Slovenian language.

### Randomization and Blinding

Through simple randomization, the participants will be divided into the intervention and control groups, thus providing all participants with the same opportunity to be selected into any group in the study [41]. The randomization process will be blinded, as this will avoid the possibility of choice bias [42,43]. Blinded group assignment will be enabled using a group assignment software, which will allow a general random selection of the individual groups. The researcher involved in the randomization process and the patients themselves will not know to which group the patients were assigned. The medical staff will be informed about the start of the research and use of the mobile app by the individuals in the intervention group. The only difference between the intervention and control groups will be the use of the *forDiabetes* [36] mobile app, which the intervention group will use from the beginning of the study until the first control visit. Both groups will receive instruction regarding type 2 diabetes and the related self-care.

### Data Collection

The initial part of the questionnaire consists of questions about the patient’s demographic data and data on the patient’s support from the medical staff. The first part of the questionnaire will cover the assessment of self-care. Self-care in patients with type 2 diabetes will be assessed using the SCODI instrument [39]. The tool consists of 40 assumptions classified into 4 dimensions: self-care maintenance (12 assumptions), self-care implementation (8 assumptions), self-care monitoring (9 assumptions), and self-confidence (11 assumptions). The instrument is assessed using a Likert scale, where a higher score represents better self-care [39]. We will also use the Brief IPQ scale [40] to assess disease perception. The questionnaire rapidly assesses disease perception and measures the patients’ cognitive and emotional representations of their disease. The questionnaire contains 8 questions graded on a 10-point scale and 1 open-ended question. There are 5 questions that assess the cognitive perceptions of the disease: consequences (point 1), timeline (point 2), personal control (point 3), treatment control (point 4), and identity (point 5); 2 questions that assess emotional representation: care (point 6) and emotions (point 8); and 1 question that estimates the comprehensibility of the disease (point 7). The assessment of causal representation is based on an open response that requires the patients to indicate the 3 most important causal factors in their disease (point 9) [40]. The nurses employed at the family medicine practices will use standardized, calibrated devices according to the guidelines to measure blood sugar, blood pressure, and HbA_1c_ levels and body weight. Data will be recorded at each control check.

The questionnaires were translated using the reverse translation method into English. This ensured the adequacy and consistency of the translated questionnaire. It was first translated from English into Slovene independently by 2 translators. One of the translators involved had expertise in nursing, and the other had the necessary knowledge of the standard English language. Thus, we obtained 2 translation versions, which were merged in the next step through review and consultations. In the last step, 2 experts with the necessary English knowledge translated a standard Slovenian questionnaire into English and combined it into a familiar form. Thus, we obtained 2 translated questionnaires in English, which we subsequently incorporated into a standard format [44,45]. The final version of this translated questionnaire will need to be preconfirmed and tested on a small sample, and the subsequent corrections will be tested on a large representative sample of respondents [46]. We will carry out the validation of the questionnaire. To analyze the reliability of the questionnaire, we will calculate Cronbach α and perform confirmatory factor analysis. Cronbach α measures intrinsic reliability among multiple elements and assesses how reliable the questionnaire responses are [47]. If the Cronbach α value is low (close to 0), it means that some or all of the items do not measure the same dimension [47-49]. The content validation process will consist of the following steps: the preparation of a content review form, selection of an expert review team, implementation of content review, review of domains and elements, determination of the rating for each item, and calculation of Content Validity Index (CVI) [50]. The content validity will be assessed by a nurse employed at the family medicine practice with knowledge of type 2 diabetes self-care. To assess the substantive validity of the questionnaire, we will use the index of substantive validity for individual statements (Item-CVI) and the entire questionnaire (Scale-level CVI). We will use a 4-point scale for evaluation [50]. Item-CVI results higher than 0.78 from 3 or more experts in assessing an individual statement represent good substantive validity [51]. Scale-level CVI results of 0.80 or more indicate good substantive validity [52].

### Inclusion Criteria

A randomized controlled trial will be conducted in family medicine practices where individuals with type 2 diabetes are treated throughout Slovenia. There are 10 regional units of the Health Insurance Institute of Slovenia, with 241 private and 656 public health institutions [53]. The research will cover 1 family medicine practice from each regional unit. According to the data from January to September 2018, there were 28,593 treatments of individuals in connection with type 2 diabetes conducted in family medicine practices [54]. This number was used to calculate the sample size, which was calculated using a sample size calculator (Raosoft) [55] that has been used in many similar health research studies [56-59]. We used a power analysis and entered the necessary data with a 95% error rate and 5% confidence level. The required minimum sample size is 380 participants. We will include individuals aged over 18 years with type 2 diabetes who have already been given treatment. Additionally, there should be no acute complications due to the consequences of type 2 diabetes in the individuals included in the study. They must use a mobile phone and a blood glucose meter during the research period.

### Statistical Analysis

The sample will be described with descriptive statistics, and the explanatory variables will be presented with frequencies and percentages. In contrast, the numerical variables will be presented with the mean value, SD, and minimum and maximum values. To test the relationship between different variables, we will use the Mann-Whitney *U* test in the case of an uneven distribution of variables or the *t* test of independent samples in an even distribution of variables. The significance level of α=.05 will test the hypotheses.

### Ethics Approval

We received permission from The Republic of Slovenia National Medical Ethics Committee (03/5R-2021) for the primary research. We will also obtain the necessary consent from all institutions and participants in a randomized controlled trial. We will obtain permits from the institutions to enter the family medicine practices to conduct the research. We will also obtain consent from the participants to participate in the study. Before starting the analysis, we received written authorization to use the mobile app *forDiabetes* [36]. We also obtained permission from the authors to use and translate the questionnaires SCODI [39] and Brief IPQ [40]. In the introductory part of the research, the research participants will be acquainted with the purpose and goal of the study, ensuring the anonymity of the participants, voluntary nature of participation, and possibility of withdrawing from participation. Participants who submit a completed questionnaire will consent to the research and use of their data. The data will be securely stored and used only for research and publication, and only the researchers involved will have access to the actual data. The data will be kept for 10 years. We will ensure the anonymity of the participants throughout the research; it will not be possible to identify a person and institution or other demographic data from the results presented. The study does not pose a risk to the participants and represents a minimal burden. We will also consider the principles of legality, fairness, proportionality, accuracy, and timeliness of the data during the research

## Results

Before the current phase of the research, we have prepared a translation of the mobile app that will be used by the participants of the intervention group, as well as more detailed instructions for using the app. We have also prepared a translation of the questionnaires in Slovene. The research results will be published in 2023.

## Discussion

### Expected Findings

Adherence to chronic management measures is essential for maintaining good health outcomes [60]. By 2045, the number of patients with diabetes will rise to approximately 629 million [61]. Mobile apps provide motivation and support for self-care in these patients [62], but there is still a lack of knowledge about their actual use [63].

Based on the described methodological process, we will perform a randomized controlled trial to determine the effectiveness of a mobile app and its impact on patients with diabetes. The research represents a unique opportunity to use and integrate mobile apps in the treatments of individuals with type 2 diabetes to solve challenges and achieve better self-care. We will enable the use of an efficient and evidence-based mobile app that is suitable for users’ needs and includes the concept of self-care in the framework of mobile health. The research will help to gain new knowledge in integrating mobile technology into the life of an individual with type 2 diabetes.

### Limitations

The main limitation is that we will only include patients with controlled and managed type 2 diabetes in the study, which means that we will examine only a specific part of the population. The study will also have only patients treated in family medicine practices, which may prevent the possibility of generalization to all people with type 2 diabetes. 

Having both control and intervention groups at the same family medicine practices may provide bias, as this will not conceal the allocation of an intervention in a randomized controlled trial. Researchers will know in which group the participants were assigned, as these participants will carry out the necessary training for the use of the mobile app and other interventions. The research will use a questionnaire to assess the participants’ self-care and perception of the disease, which can provide socially expected and desirable answers.

Our study contains the following assumptions. People with type 2 diabetes have deviations or shortcomings in each concept of self-care. The app will affect a patient’s self-care with type 2 diabetes and other critical clinical measures. Self-care and disease perception are measurable and that the translated questionnaire will be helpful in our research. Medical professionals, employees in family medicine practices, and individuals with type 2 diabetes will be willing to participate in the study. People with type 2 diabetes will actively use the mobile app and monitor their health through data entry. The results expected during the study are changes in self-care; disease perception; blood sugar, blood pressure, and HbA_1c_ levels; and measured body weight.

## Abbreviations

Brief IPQ: Brief Illness Perception Questionnaire
CVI: Content Validity Index
HbA_1c_: glycated hemoglobin
SCODI: Self-Care of Diabetes Inventory
